# First-Principles Design Rules for Selective Room-Temperature Gas Sensing in Transition-Metal-Doped MoS_2_

**DOI:** 10.1021/acsami.5c23014

**Published:** 2026-02-21

**Authors:** Maciej J. Szary

**Affiliations:** Institute of Physics, 49632 Poznan University of Technology, ul. Piotrowo 3, 61-138 Poznan, Poland

**Keywords:** 2D materials, defect engineering, air pollutants, structure−activity relationships, DFT modeling

## Abstract

Substitutional doping provides a practical and scalable strategy to tune the surface reactivity of transition-metal dichalcogenides (TMDs) for chemical sensing, yet the underlying relationships between dopant identity and adsorption behavior remain poorly understood. Here, first-principles calculations are employed to examine the effects of substituting molybdenum in monolayer MoS_2_ with transition metals from groups 4 (Zr, Hf), 5 (Nb, Ta), 7 (Tc, Re), and 10 (Pd, Pt). These dopants, which themselves form stable TMD monolayers, incorporate effectively into the MoS_2_ lattice—consistent with their demonstrated feasibility via chemical vapor deposition (CVD). While the adsorption of NH_3_, CO_2_, SO_2_, and NO_2_ remains governed by physisorption, distinct dopant-dependent selectivity emerges. Group 4, 5, and 10 dopants induce negligible modulation of adsorption and charge transfer, whereas group 7 dopants markedly enhance the NO_2_ binding and electron exchange. The resulting carrier concentration changes reach up to 4 orders of magnitude higher than in pristine MoS_2_ at sub-ppm analyte levels and room temperature, enabling potential ppb-level NO_2_ detection with minimal cross-sensitivity to SO_2_. These results establish transition-metal substitution as a chemically coherent and experimentally viable route for selectively boosting the sensitivity of MoS_2_-based gas sensors toward oxidizing, polar analytes such as NO_2_.

## Graphical abstract

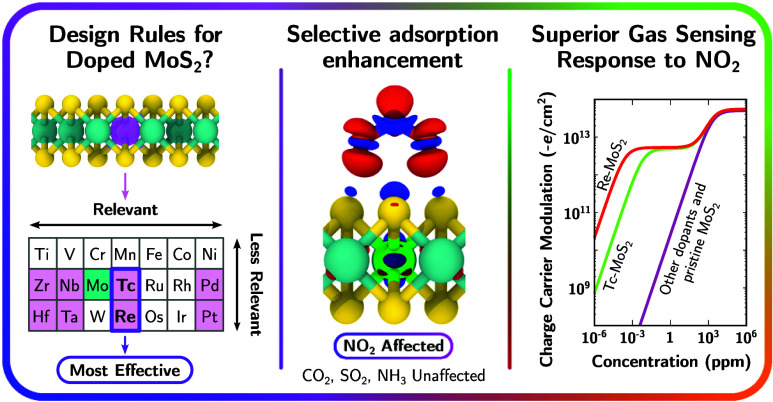

## 1 Introduction

Reliable detection and monitoring of trace gases are increasingly critical amid growing environmental and public health concerns.^1–4^ Anthropogenic emissions of toxic and greenhouse gases such as NO_*x*_, CO_*x*_, SO_2_, and NH_3_ affect air quality, climate dynamics, and human well-being. Consequently, there is an urgent demand for real-time, low-cost, and energy-efficient sensing platforms capable of operating under ambient conditions for industrial safety, smart cities, and portable environmental diagnostics.^5,6^ Analytical methods based on spectroscopic or mass spectrometric detection offer excellent selectivity and sensitivity but are often expensive, power-intensive, and unsuitable for field deployment.^7^ In contrast, conventional metal-oxide semiconductor sensors are compact and cost-effective but typically require elevated operating temperatures and exhibit cross-sensitivity and poor selectivity, particularly in humid or chemically complex environments.^8,9^ Low-dimensional nanomaterials present a compelling alternative, enabling ultrathin, flexible, and low-power sensing platforms compatible with emerging electronic and photonic technologies.^10–13^ However, their practical implementation remains constrained by an incomplete understanding of surface chemistry, which complicates the rational optimization of sensitivity, selectivity, and long-term stability under ambient conditions.

Two-dimensional (2D) transition-metal dichalcogenides (TMDs) have emerged as one of the most promising material classes for room-temperature chemical sensing, owing to their unique structural, electronic, and chemical characteristics.^14–16^ Among them, molybdenum disulfide (MoS_2_) is widely regarded as the prototypical TMD because of its thermodynamic stability, well-defined semiconducting behavior, and scalable synthesis in large-area monolayers.^17^ Its layered crystal structure—comprising covalently bonded S–Mo–S sheets held together by van der Waals (vdW) forces—provides a high surface-to-volume ratio where every atom participates in surface interactions. The nonbonding lone-pair orbitals of sulfur facilitate physisorption-driven charge transfer with adsorbed species,^18^ enabling even weakly bound molecules to modulate MoS_2_ conductivity. This property makes MoS_2_-based devices particularly responsive to strong electron acceptors such as NO_2_,^19–21^ underscoring their potential in environmental monitoring and chemical sensing applications.

Despite these advantages, pristine TMDs exhibit limited sensitivity toward nonpolar or electron-donor molecules such as CO_2_ and NH_3_ and poor selectivity among similar electron-accepting species (e.g., NO_2_ and SO_2_).^22^ These intrinsic limitations stem from the largely homogeneous electronic landscape of the basal plane, which lacks specific binding sites that can differentiate adsorbates of comparable chemical nature. To overcome these challenges, several modification strategies—such as surface functionalization,^23–27^ defect engineering,^28–30^ and chemical doping^31–38^ —have been explored to tune adsorption characteristics. However, most prior studies have focused narrowly on quantifying improvements in the sensing performance toward targeted species rather than elucidating the underlying chemical mechanisms or establishing generalizable design principles linking dopant chemistry to trends in adsorption phenomena.

To address these gaps, this work presents a systematic density functional theory (DFT) investigation aimed at identifying design principles for optimizing TMD-based gas sensors through transition-metal (TM) substitutional doping (Figure 1). MoS_2_ serves as the model substrate due to its well-characterized structure and prevalence in gas sensing research. The selected dopants—Zr, Nb, Tc, Pd, Hf, Ta, Re, and Pt—were chosen based on their demonstrated ability to form stable layered TMD monolayers,^39^ ensuring both structural compatibility and synthetic feasibility under chemical vapor deposition (CVD) conditions.^40–46^ This substitutional approach preserves the structural integrity of the MoS_2_ lattice while introducing localized electronic perturbations that can selectively enhance or suppress gas–surface interactions.

**Figure 1:**
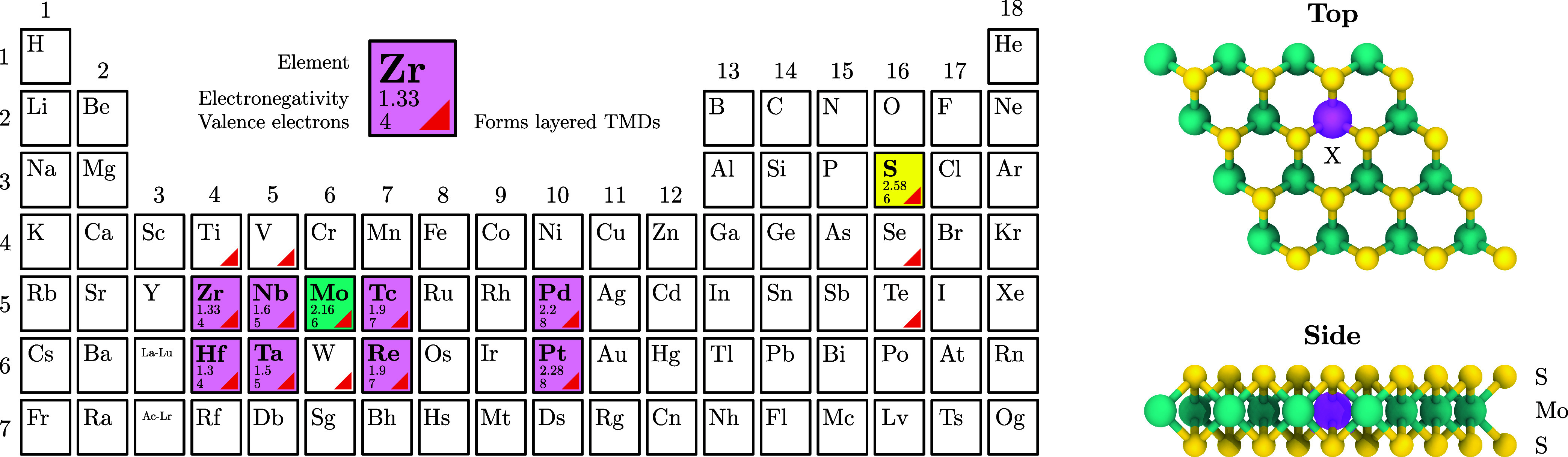
Schematic representation of sulfur (yellow) and molybdenum (teal) atoms forming a stable MoS_2_ monolayer with hexagonal symmetry. The transition-metal atoms are coordinated by chalcogens in a characteristic sandwich-type structure. Transition-metal dopants (pink) substitute at the molybdenum sites, and their positions relative to Mo are highlighted in the periodic table.

The dopants were further selected to represent a chemically diverse set with varying valence electron counts relative to Mo: group 4 (Zr, Hf) having two fewer electrons, group 5 (Nb, Ta) one fewer electron, group 7 (Tc, Re) one more electron, and group 10 (Pd, Pt) four more electrons. This controlled variation establishes a tunable electronic landscape that might modulate MoS_2_ responses to donor and acceptor molecules in distinct ways, thereby enhancing both sensitivity and selectivity. To capture this capacity for distinct adsorption behaviors, the study considers four environmentally relevant gases—NH_3_, CO_2_, SO_2_, and NO_2_—spanning diverse donor–acceptor characteristics. The insights derived from this investigation provide a rational framework for dopant selection and contribute to the broader understanding of how transition-metal chemistry governs adsorption phenomena in TMD-based gas sensors.

## 2 Methods

### 2.1 Computational Setup

All DFT calculations were carried out using the Quantum ESPRESSO package.^47–49^ The electronic structure was described using plane-wave basis sets in conjunction with projector augmented wave (PAW) pseudopotentials. Kinetic energy cutoffs were set to 60 Ry for the wave functions and 600 Ry for the charge density. Scalar relativistic effects and nonlinear core corrections were included in all pseudopotentials, and spin polarization was considered consistently between calculations. The Brillouin zone was sampled using a 4 × 4 × 1 Monkhorst–Pack grid.^50^ Exchange–correlation (XC) effects were described using the Perdew–Burke–Ernzerhof (PBE) generalized gradient approximation (GGA)^51,52^ complemented by Grimme’s D3 dispersion correction.^53,54^ This combination has been shown to yield highly reliable results for both structural and interfacial properties of TMDs, even in comparison with fully nonlocal XC functionals.^55^ The robustness of the PBE + D3 approach is further corroborated by numerous studies demonstrating its accuracy in capturing physisorption and charge-transfer phenomena in two-dimensional materials.^18,22,56–60^ Partial atomic charges were determined via Löwdin population analysis applied to the valence states;^61^ see the Supporting Information for details and for cross-validation against the Bader charge analysis.^62^ Vibrational frequencies were calculated by using density functional perturbation theory (DFPT).

### 2.2 Structural Models

Pristine monolayer MoS_2_ was modeled by using a 4 × 4 supercell representation. Transition-metal doping was introduced by substituting a single Mo atom with one of the selected dopant species (Figure 1). Convergence analyses across supercells ranging from 2 × 2 to 7 × 7 indicated that size effects are negligible for supercells larger than 4 × 4 (see the Supporting Information for details), consistent with previous adsorption studies.^33,35,63–67^ Adsorbates were positioned on the top surface of the doped monolayer to simulate experimental exposure conditions, thereby inducing a small out-of-plane dipole moment. To eliminate spurious interactions between periodic images, a dipole correction was applied^68^ within a vacuum region of approximately 20 Å. All structures were fully relaxed using the Broyden–Fletcher–Goldfarb–Shanno (BFGS) algorithm^69–72^ until the residual forces were below 10^–4^ Ry/au and total energy changes were less than 10^–5^ Ry between iterations.

## 3 Results and Discussion

### 3.1 Dopant Incorporation into the MoS_2_ Lattice

Before assessing the effects of transition-metal substitution on the local surface reactivity of MoS_2_, it is essential to first examine how different dopants are incorporated into the host lattice. Structural integrity and bonding configuration are key indicators of dopant compatibility: minimal lattice distortion ensures thermodynamic stability^73^ and enables meaningful comparisons of reactivity trends that arise primarily from dopant identity rather than structural changes. In contrast, extensive lattice disruption—such as broken Mo–S or TM–S bonds—can create undercoordinated sites with potentially enhanced catalytic activity but reduced environmental stability,^74^ deviating from the conventional sensing framework of TMDs.^22^

Figure 2 presents the optimized atomic geometries of pristine and doped MoS_2_ monolayers. The results reveal that the overall impact of doping on the atomic structure is generally modest, with systematic variations observed across periodic identities of the dopants. Group 4, 5, and 7 elements readily substitute Mo atoms within the lattice while preserving the local trigonal prismatic coordination environment. Specifically, group 7 dopants (Tc and Re) induce a slight contraction of the lattice around the substitution site (compare Figure 2c,2d,2h), whereas group 5 dopants (Nb and Ta) cause a minor local expansion (Figure 2b,2g). Group 4 dopants (Zr and Hf) exhibit the largest structural relaxation, with TM–S bond lengths increasing by approximately 0.1 Å relative to that of pristine MoS_2_ (Figure 2a,2f).

**Figure 2:**
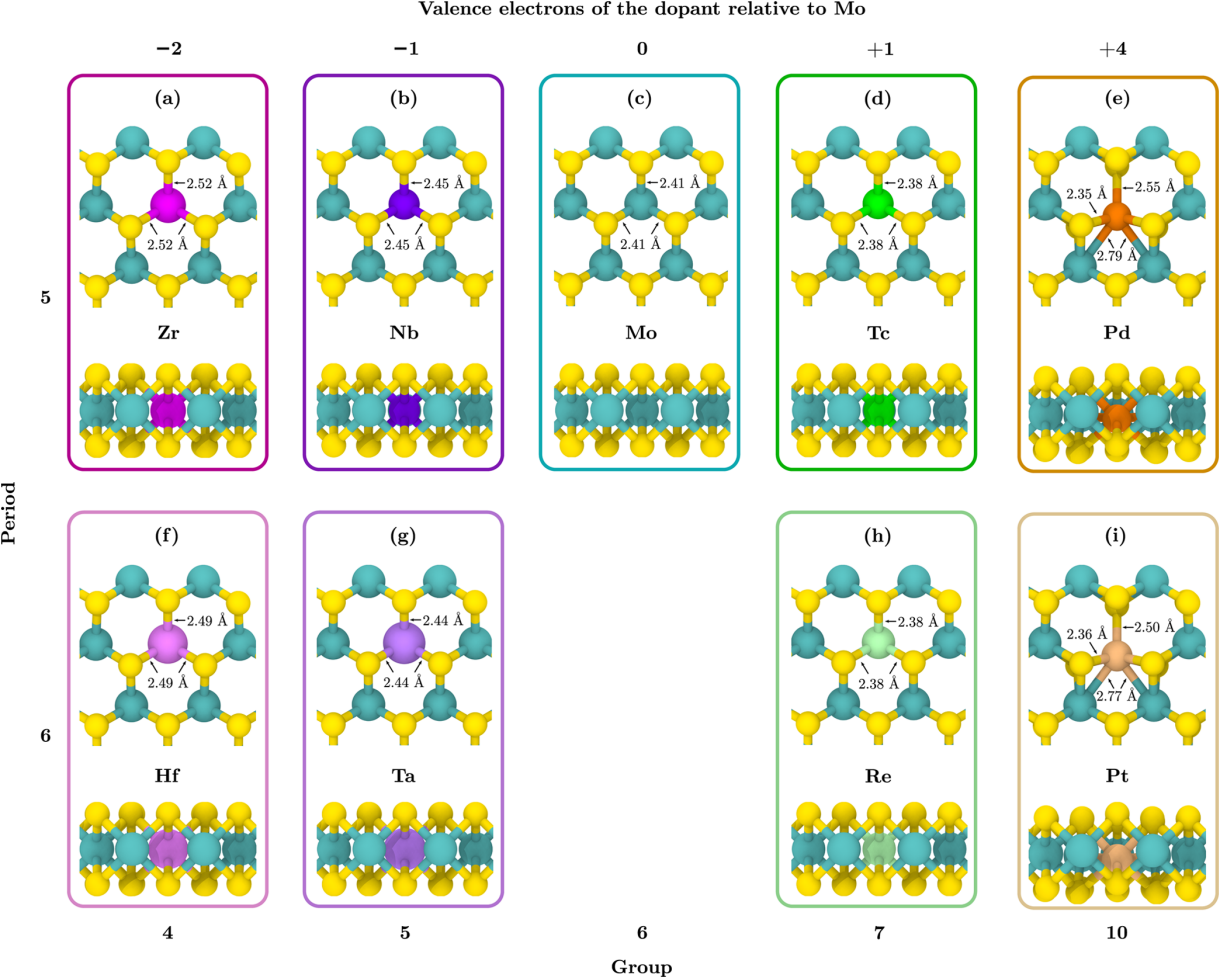
Optimized atomic structures of (a) Zr–MoS_2_, (b) Nb–MoS_2_, (c), MoS_2_, (d) Tc–MoS_2_, (e) Pd–MoS_2_, (f) Hf–MoS_2_, (g) Ta–MoS_2_, (h) Re–MoS_2_, and (i) Pt–MoS_2_.

In contrast, group 10 dopants (Pd and Pt) exert a markedly different influence on the local geometry (Figure 2e,2i). Although both elements can formally occupy the Mo substitutional site, these configurations are only metastable, lying 0.6 eV (Pd) and 0.9 eV (Pt) higher in energy compared with reconstructed geometries. In the latter, the dopant atoms shift off-center toward adjacent Mo atoms, positioning themselves within the sum of their covalent radii. This displacement perturbs the coordination of nearby sulfur atoms—shortening TM–S bonds on one side while stretching or partially breaking them on the opposite side. The resulting asymmetry leads to localized lattice distortion and a partial loss of the pristine trigonal prismatic symmetry.

Overall, these results indicate that dopants from groups 4, 5, and 7 incorporate into the MoS_2_ lattice in a manner similar to that of Mo, giving rise to predictable p- and n-type characteristics that reflect their differing valence electron counts. Correspondingly, acceptor- and donor-like states appear near the valence and conduction band edges, respectively (see the Supporting Information for details and DOS plots). In contrast, group 10 dopants cause pronounced local reconstruction, introducing both structural and electronic complexity, while contributing additional electrons to the monolayer. As a result, Pd- and Pt-doped MoS_2_ systems are expected to exhibit adsorption trends distinct from those of other electron-rich dopants such as Tc and Re.

### 3.2 Microscopic Adsorption Behavior

Following the evaluation of the incorporation of the dopant into the MoS_2_ lattice, the next step is to examine how these modifications affect molecular adsorption behavior. To this end, the adsorption of four representative gas species—NH_3_, CO_2_, SO_2_, and NO_2_—was systematically investigated across all considered substrates. Each molecule was placed at four distinct adsorption sites on the surface (as illustrated in Figure 3), with two molecular orientations evaluated per site. In total, 288 adsorption configurations were analyzed (2 orientations × 4 adsorption sites × 4 adsorbates × 9 substrates), enabling a comprehensive assessment of dopant-dependent trends in adsorption energetics and binding characteristics.

**Figure 3:**
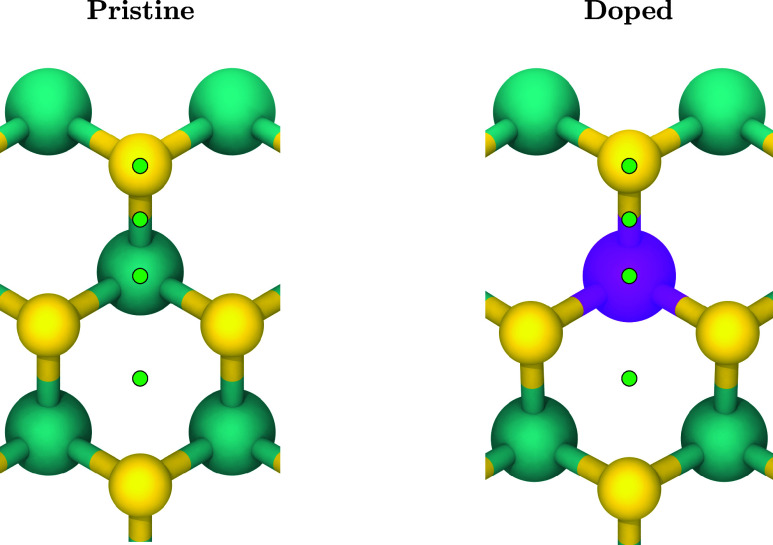
Surface sites considered for probing adsorption configurations on pristine and doped MoS_2_. Individual sites are indicated by green dots.

The lowest-energy adsorption configurations identified for each molecule on all of the investigated monolayers are presented in Figure 4. Overall, the results indicate that substitutional doping has a relatively minor influence on the qualitative nature of adsorption and its configuration geometry. Across nearly all systems, the adsorbates maintain similar orientations and comparable molecule–surface separations, consistent with physisorption-dominated interactions. This uniformity suggests that, despite local electronic perturbations introduced by the dopants, the overall interaction landscape of the MoS_2_ surface remains largely governed by van der Waals forces rather than chemisorption.

**Figure 4:**
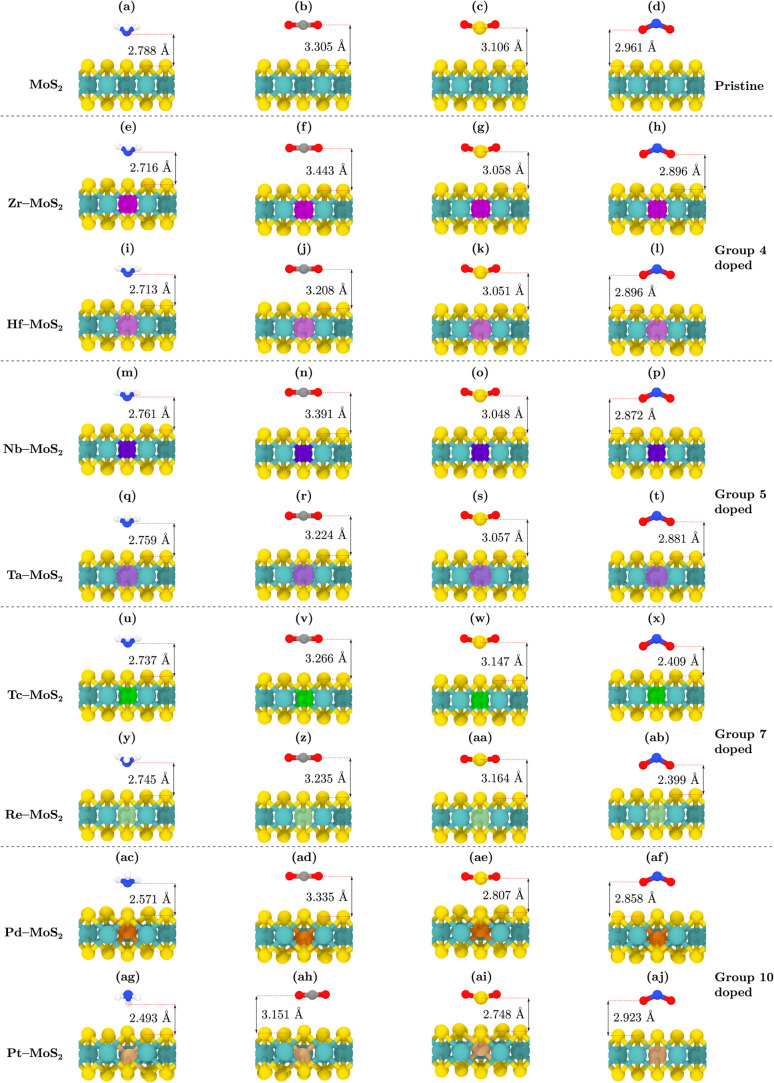
Optimized atomic structures of the lowest-energy configurations for the adsorption of (far left) NH_3_, (center left) CO_2_, (center right) SO_2_, and (far right) NO_2_ molecules on (a–d) pristine and (e–aj) doped MoS_2_ monolayers.

For ammonia, the molecule typically adsorbs with its nitrogen atom facing the monolayer and hydrogen atoms oriented away from the surface, relaxing at an equilibrium distance of approximately 2.7 Å for both pristine and doped substrates. A slight reduction in adsorption height to about 2.5 Å is observed for monolayers doped with group 10 elements (Pd and Pt), suggesting a modest enhancement in the interaction strength. Notably, in the case of Pt-doped MoS_2_, the NH_3_ molecule adopts an inverted configuration with the nitrogen atom pointing away from the surface (Figure 4ag); however, the energy difference between the two orientations remains small, on the order of 20 meV.

Carbon dioxide consistently adsorbs in a flat, horizontally aligned configuration parallel to the MoS_2_ surface, with molecule–sheet separations ranging from 3.15 to 3.44 Å across the various monolayers. This uniform geometry reflects the nonpolar nature of CO_2_ and its limited charge-transfer capability, indicating that the examined doping strategies exert only a minimal influence on its adsorption characteristics.

Sulfur dioxide also favors a quasiplanar configuration, although a slight molecular tilt is observed depending on the dopant species. The sulfur atom tends to shift marginally toward the substrate, yielding equilibrium separations between 3.05 and 3.16 Å. As in the case of NH_3_, group 10 dopants strengthen the interaction, reducing the adsorption distance to approximately 2.8 Å (see Figure 4ae,4aj). These geometric trends suggest a modest dopant-induced polarization effect that slightly enhances the van der Waals coupling of SO_2_ to the surface.

In contrast, nitrogen dioxide exhibits a distinct vertical adsorption geometry with the oxygen atoms directed toward the monolayer and the nitrogen atom pointing outward. For pristine MoS_2_ and systems doped with group 4, 5, and 10 elements, the NO_2_ molecule relaxes at a distance of approximately 2.9 Å above the surface. However, for group 7 dopants (Tc and Re), this distance decreases notably to around 2.4 Å (Figure 4x,ab), indicating stronger molecule–substrate coupling. The enhanced interaction likely arises from partial charge transfer facilitated by the higher valence electron count of these dopants, consistent with their effective n-type doping behavior within the monolayers (see the Supporting Information for DOS plots).

To quantitatively assess how substitutional doping modifies the adsorption behavior of MoS_2_, two key descriptors were evaluated: the adsorption energy (*E*_ads_), which measures the strength of binding between the adsorbate and the substrate, and the charge transfer (Δ*Q*_ads_), which characterizes the degree of electronic exchange induced by their interaction. Together, these parameters provide complementary insight into how dopant-induced electronic perturbations influence the physisorption strength and sensing response.

The adsorption energy is defined as

$$E_{\mathrm{ads}}=E_{\mathrm{tot}}\left(\mathrm{mol.}@\mathrm{TMD}\right)-E_{\mathrm{tot}}\left(\mathrm{bare}\;\mathrm{TMD}\right)-E_{\mathrm{tot}}(\mathrm{free}\;\mathrm{mol.})$$ 1

where *E*_tot_(mol.@TMD) is the total energy of the relaxed adsorbate–substrate complex, *E*_tot_(bare TMD) corresponds to the clean monolayer (either pristine or doped), and *E*_tot_(free mol.) refers to the isolated gas-phase molecule. In this convention, more negative *E*_ads_ values indicate stronger binding interactions between the adsorbate and the surface. To ensure practical recovery at room temperature, *E*_ads_ should not fall below −1.0 eV.^75^ The charge transfer is expressed as

2 $$\Delta Q_{\mathrm{ads}}=Q_{\mathrm{ads}}\left(\mathrm{mol.}\right)-Q_{\mathrm{gas}}\left(\mathrm{mol.}\right)$$

where *Q*_gas_(mol.) and *Q*_ads_(mol.) denote the net charges on the molecule before and after adsorption, respectively, as obtained from Löwdin population analysis. Negative values of Δ*Q*_ads_ indicate electron gain by the adsorbate (i.e., charge withdrawal from the substrate), whereas positive values correspond to electron donation to the monolayer.

The calculated adsorption energies and charge transfers for the lowest-energy configurations are summarized in Figure 5. Panel (a) illustrates how adsorption parameters vary with the chemical identity of the dopant, while panels (b) and (c) plot adsorption energy and charge transfer, respectively, as functions of the dopant’s valence electron count relative to Mo.

**Figure 5:**
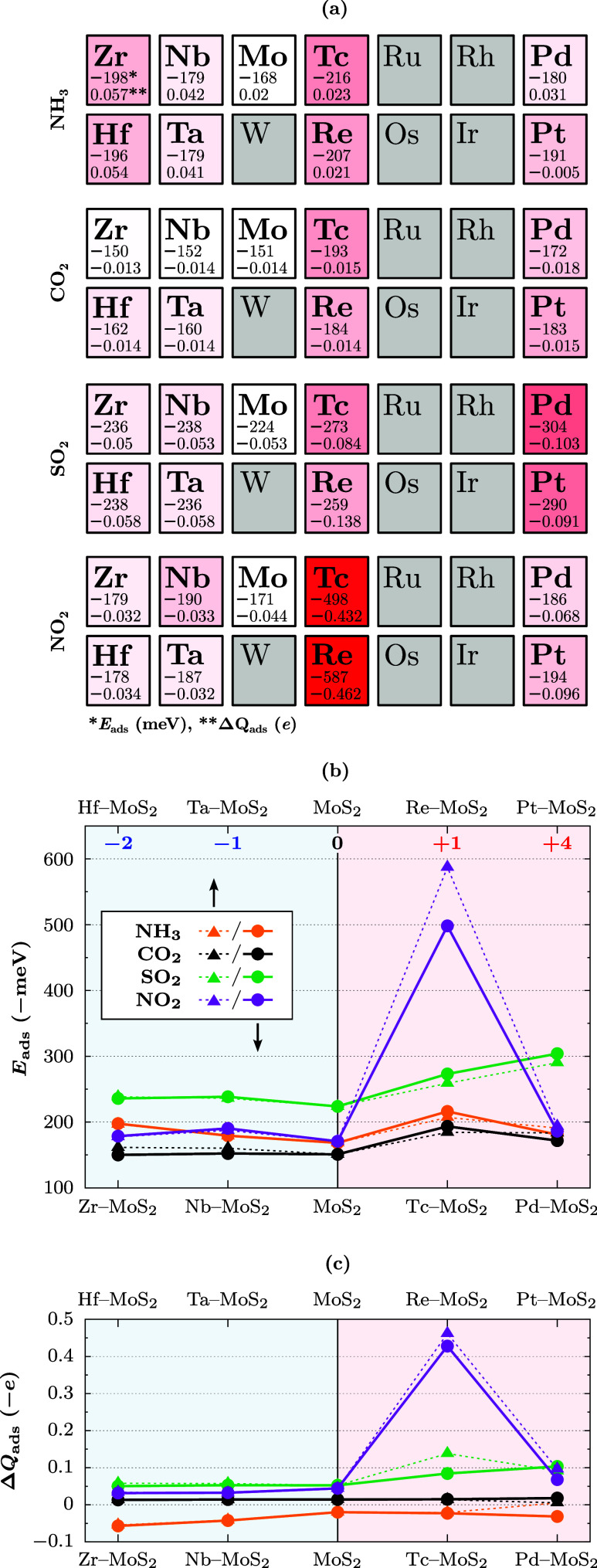
(a) Adsorption parameters mapped according to the positions of transition-metal elements in the periodic table for pristine and doped MoS_2_, with corresponding trends in (b) adsorption energies and (c) charge transfers plotted across the substrate types.

Overall, the results confirm that adsorption across all systems remains within the characteristic range of physisorption, yet doping induces notable species-selective variations in interaction strength and charge transfer. Adsorption on pristine MoS_2_ is generally the weakest for all molecules, while substitutional doping consistently enhances interaction strength—although not symmetrically between electron-deficient and electron-rich dopants. Differences between period 5 and 6 dopants within the same group are generally minor, suggesting that the dominant trends arise predominantly from the dopant’s valence configuration rather than its atomic size.

For NH_3_, a strong electron donor, adsorption is systematically enhanced by p-type dopants from groups 4 and 5. The effect is most pronounced for Zr-doped MoS_2_, where *E*_ads_ increases in magnitude from −168 to −198 meV, accompanied by a rise in Δ*Q*_ads_ from 0.020 to 0.057 e. Interestingly, although group 7 dopants (Tc and Re) are n-type, they also strengthen adsorption, albeit without a comparable increase in charge transfer; yielding, for example, *E*_ads_ = −216 meV and Δ*Q*_ads_ = 0.023 e for Tc–MoS_2_. In contrast, group 10 dopants exert a weaker influence than those from group 7, likely due to differences in their local bonding environments. Overall, these effects remain small, indicating the limited sensitivity of NH_3_ to the electronic perturbations of the substrate.

Doping exerts a similarly limited influence on the CO_2_ adsorption. On pristine MoS_2_, the molecule exhibits weak physisorption with *E*_ads_ = −151 meV and negligible charge transfer (Δ*Q*_ads_ = −0.014 e). Group 4 and 5 dopants induce virtually no change, consistent with the poor donor character of CO_2_. Group 7 elements enhance adsorption slightly, reaching *E*_ads_ = −193 meV for Tc–MoS_2_, while group 10 dopants again display minimal influence, likely due to bonding reconfigurations involving Pd and Pt. Overall, CO_2_, being nonpolar and chemically inert, remains largely unresponsive to electronic modulation through substitutional doping.

For SO_2_, a polar and stronger electron-accepting molecule, doping induces more pronounced effects. Adsorption on pristine MoS_2_ yields *E*_ads_ = −224 meV and Δ*Q*_ads_ = −0.043 e. Group 4 and 5 dopants exert a modest influence, with Hf-doped MoS_2_ reaching *E*_ads_ = −238 meV and Δ*Q*_ads_ = −0.058 e. In contrast, dopants from groups 7 and 10 notably enhance the interaction, particularly for Pd-doped MoS_2_, which exhibits *E*_ads_ = −304 meV and Δ*Q*_ads_ = −0.103 e. These results indicate that electron-rich dopants can promote polarization and charge exchange with electron-accepting species.

The most pronounced dopant effects are observed for NO_2_, the strongest electron acceptor in the studied set. On pristine MoS_2_, the molecule binds weakly (*E*_ads_ = −171 meV, Δ*Q*_ads_ = −0.044 e). Group 4 and 5 dopants lead to only modest changes (*E*_ads_ = −190 meV, Δ*Q*_ads_ = −0.033 e for Nb–MoS_2_), whereas group 7 dopants induce a significant increase in both adsorption energy and charge transfer. For Tc- and Re-doped systems, *E*_ads_ reaches −498 and −587 meV, with Δ*Q*_ads_ values of −0.432 and −0.462 e, respectively, indicating substantially strengthened physisorption potentially approaching the weak chemisorption regime. In contrast, group 10 dopants yield a markedly smaller enhancement, likely due to their reconstructed bonding configurations that locally suppress charge redistribution within the lattice.

To better understand the origin of the adsorption enhancements observed for SO_2_ and NO_2_, we further examined whether doping alters the fundamental interaction mechanism or merely strengthens the existing one. For this purpose, we evaluated the charge density difference defined as

3 Δρ=ρ(mol.@TMD)−ρ(bareTMD)−ρ(freemol.)

where ρ denotes the total pseudoelectron density.

Figure 6 visualizes these charge redistributions as isosurface maps, with red regions representing electron accumulation and blue regions representing electron depletion. For comparison, we considered pristine MoS_2_, an p-type system doped with a group 5 element (Nb–MoS_2_), where adsorption modulation is minimal, and n-type systems doped with group 7 elements (Tc–MoS_2_ and Re–MoS_2_), where the adsorption enhancement was most pronounced.

**Figure 6:**
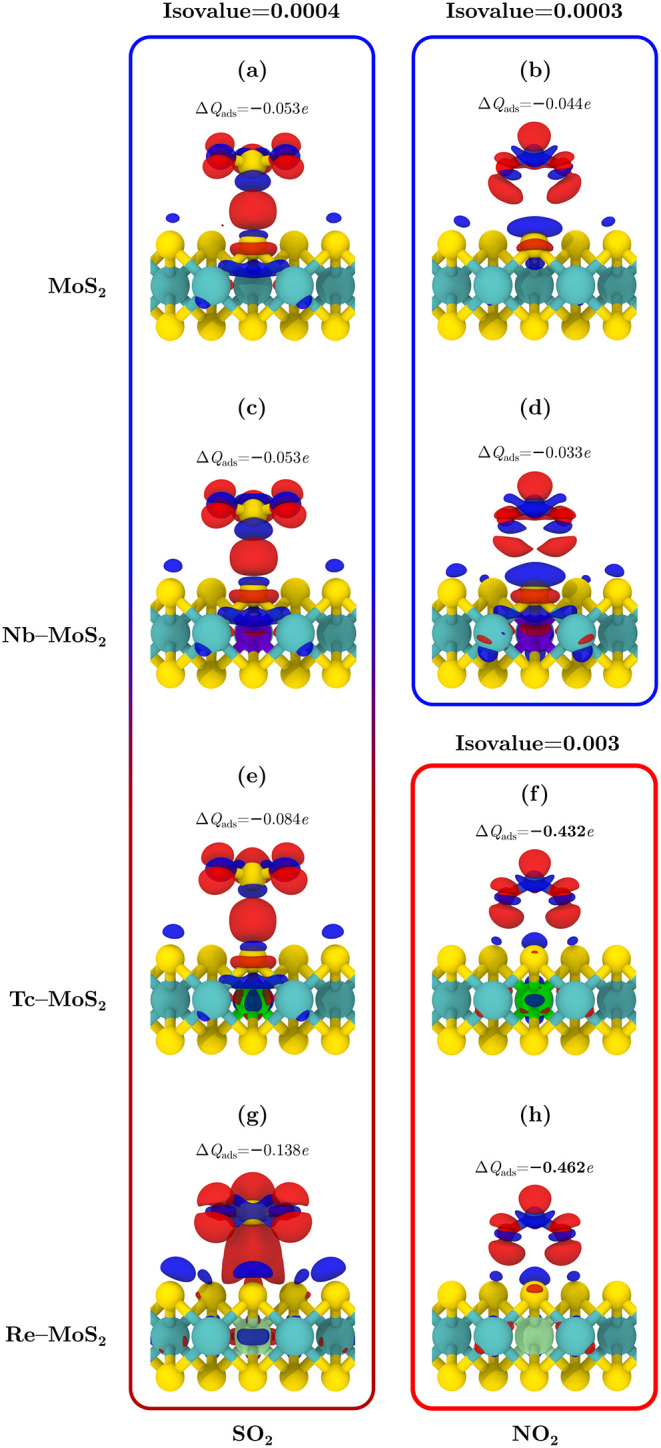
Charge density differences arising from molecule–sheet interactions on (a, b) pristine and (c–h) doped MoS_2_. Regions of charge accumulation are colored red, while regions of charge depletion are shown in blue.

For SO_2_, the charge redistribution patterns remain largely consistent across all examined substrates, regardless of the dopant type. In the case of Nb–MoS_2_, the charge rearrangement closely mirrors that of pristine MoS_2_, reflecting the negligible change in charge transfer (compare Figure 6a,6c). For Tc- and Re-doped systems (Figure 6e,g), the contours become slightly more extended, consistent with the increased charge transfer, yet the overall spatial distribution of the charge accumulation and depletion regions remains essentially unchanged.

A similar trend is observed for NO_2_. The charge redistribution pattern for Nb–MoS_2_ (Figure 6d) is nearly identical to that on pristine MoS_2_ (Figure 6b), indicating that doping does not alter the interaction topology. For Tc–MoS_2_ and Re–MoS_2_ (Figure 6f,h), the magnitude of charge transfer is significantly larger, but the spatial features of electron accumulation and depletion closely follow those in the weaker physisorption cases.

Overall, these results demonstrate that transition-metal doping does not qualitatively change the adsorption mechanism. Instead, it enhances the strength of the existing physisorptive interaction by modulating the electronic availability of the substrate, leading to a quantitatively stronger but chemically analogous adsorption behavior.

### 3.3 Macroscopic Influence on Sensing

The results discussed in Section 3.2 demonstrate that substitutional transition-metal doping can substantially modulate adsorption energetics and charge transfer at the MoS_2_ surface, particularly for polar and electron-accepting species such as SO_2_ and NO_2_. However, these microscopic effects alone do not directly determine the sensor performance. In practical applications, a key question is whether the observed changes in adsorption strength translate into measurable differences in surface coverage under realistic environmental conditions, namely, at room temperature and trace analyte concentrations.

To bridge this microscopic–macroscopic connection, we evaluate the equilibrium surface coverage of adsorbate molecules on pristine and doped sites using the Langmuir adsorption model. This approach provides a simple yet physically meaningful description of how adsorption energy influences the fraction of occupied surface sites under given thermodynamic conditions.^22,76^ Within this framework, the coverage θ is expressed as

4 $$\theta =\frac{\frac{p}{p_{0}}\;\exp\left(\frac{\Delta G^{‡}}{\mathrm{kT}}\right)}{1+\frac{p}{p_{0}}\;\exp\left(\frac{\Delta G^{‡}}{\mathrm{kT}}\right)}$$

where *p* is the partial pressure of the analyte, *p*_0_ is the standard atmospheric pressure, *k* is the Boltzmann constant, and *T* is the absolute temperature. Δ*G* ^‡^ represents the Gibbs activation energy for desorption and corresponds to either the pristine or doped site, depending on the system under consideration (see the Supporting Information for calculation details).

In this model, more exothermic adsorption (i.e., larger negative *E*_ads_) leads to higher equilibrium coverage, while elevated temperatures promote desorption and reduce θ. By construction, θ ranges from 0 to 1, corresponding respectively to fully vacant and fully occupied adsorption sites.

The resulting coverage–concentration relationships are presented in Figure 7. Panels (a) and (b) illustrate the equilibrium fractional coverage for SO_2_ and NO_2_ at 300 K as a function of analyte concentration, comparing pristine MoS_2_ with representative doped systems. For SO_2_, the curves in Figure 7a reveal a modest but systematic enhancement in site occupancy upon doping, consistent with the moderate increases in adsorption energy reported earlier. The concentration required to achieve half-coverage decreases from approximately 100 ppm for pristine MoS_2_ to around 20–40 ppm for Re- and Tc-doped systems, suggesting an improved low-level adsorption response while maintaining reversible physisorption behavior.

**Figure 7:**
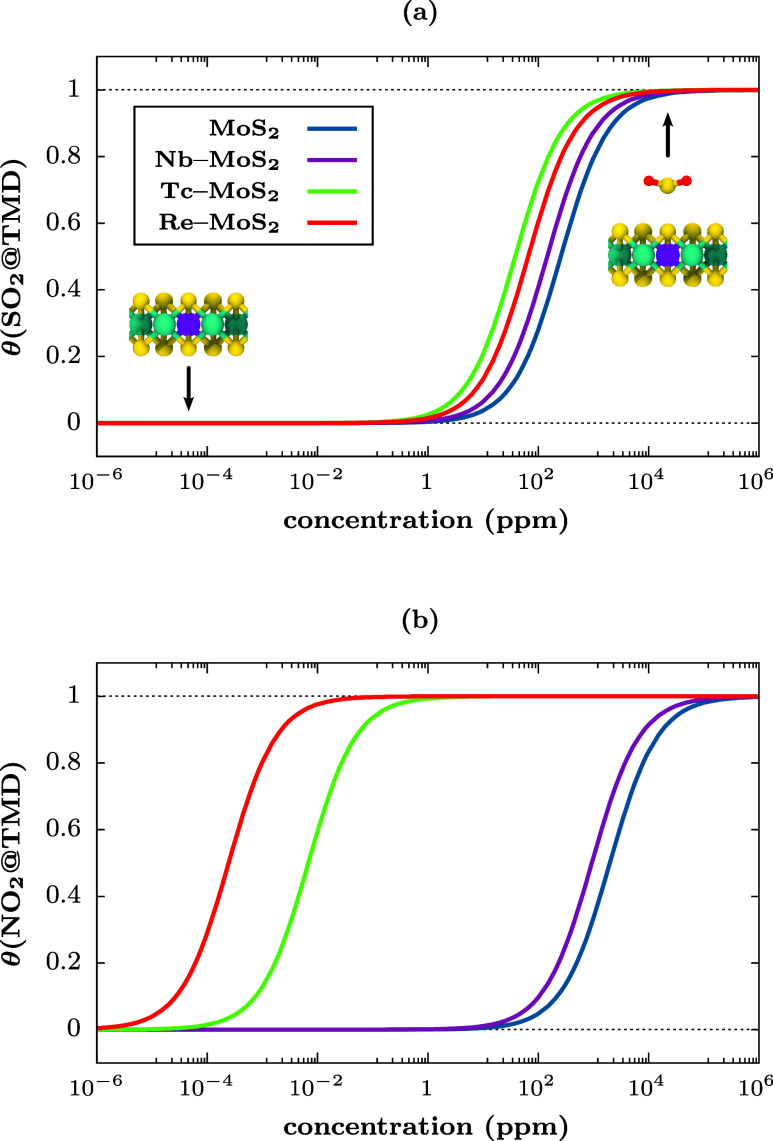
Coverage of pristine and doped MoS_2_ sites upon the adsorption of (a) SO_2_ and (b) NO_2_ at 300 K.

By contrast, the coverage behavior for NO_2_ shown in Figure 7b demonstrates a much more pronounced effect. Owing to the substantially stronger adsorption of group 7 dopants, Re–MoS_2_ and Tc–MoS_2_ achieve near-saturation coverage even at sub-ppm concentrations, indicating strong potential for high sensitivity toward oxidizing species. Meanwhile, the weaker adsorption on Nb– and pristine MoS_2_ leads to measurable coverage only above 20 ppm.

Building on the coverage analysis, these results highlight that substitutional doping modulates not only the adsorption strength but also the fraction of surface sites occupied under ambient conditions. Since each adsorbed molecule can act as a localized charge donor or acceptor, variations in surface coverage directly translate into measurable changes in the carrier concentration of the underlying monolayer.

To quantify this effect, we evaluate the change in carrier concentration (δ*n*) for both pristine and doped monolayers, using the approach established previously.^75^ For pristine TMDs (see Figure 8a), δ*n* is obtained as the product of molecular surface density and the charge transfer per molecule, yielding

5 $$\delta n_{\mathrm{pri}}=-\frac{\theta_{\mathrm{pri}}}{A}\Delta Q_{\mathrm{pri}}$$

where θ_pri_ and Δ*Q*_pri_ denote the site coverage and charge transfer for pristine MoS_2_, respectively. Here, *A* represents the area per adsorption site, corresponding to one molecule per 1 × 1 surface cell, while the negative sign ensures the correct direction of charge flow between the adsorbate and substrate.

**Figure 8:**
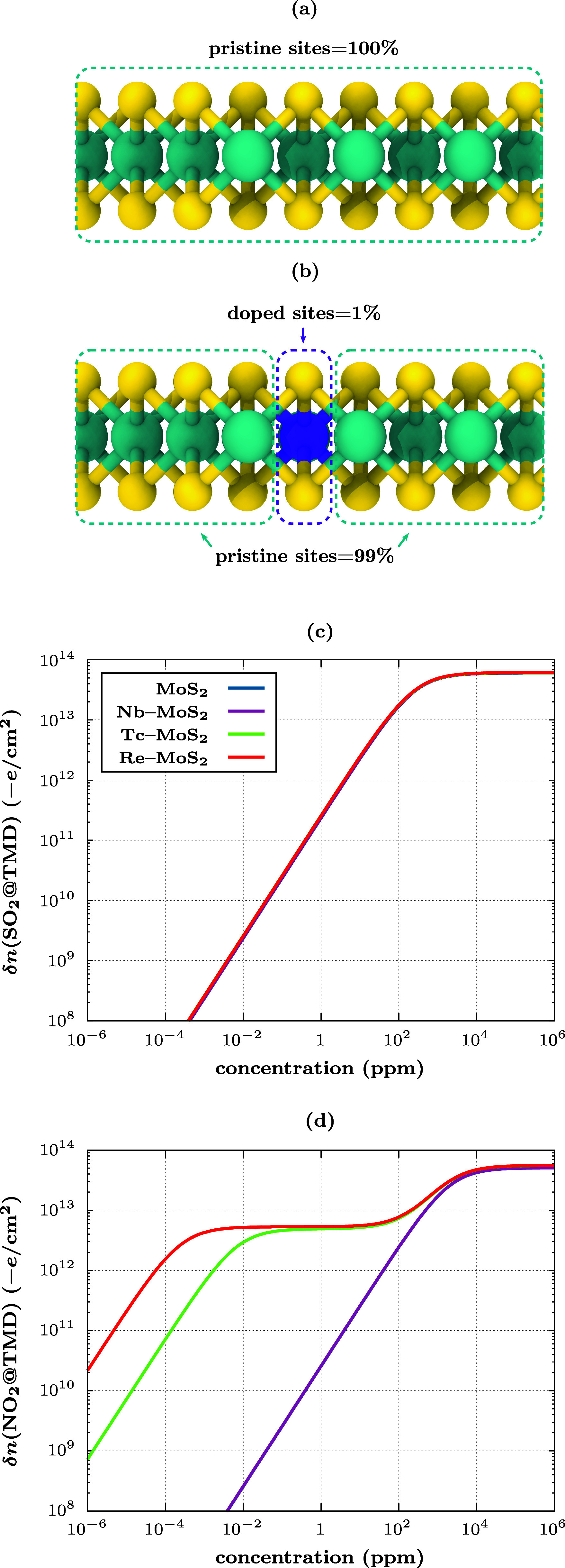
Adopted adsorption site distribution on the surface of (a) pristine and (b) doped MoS_2_ monolayers. Panels (c, d) show the corresponding variations in carrier concentration upon exposure to different concentrations of SO_2_ and NO_2_, respectively, at 300 K.

For doped monolayers, the total carrier concentration change must account for the contributions of both pristine and dopant-associated regions (Figure 8b). This leads to the composite expression

6 $$\delta n_{\mathrm{dop}}=-\left[\frac{\left(1-\chi \right)\theta_{\mathrm{pri}}}{A}\Delta Q_{\mathrm{pri}}+\frac{\chi \theta_{\mathrm{dop}}}{A}\Delta Q_{\mathrm{dop}}\right]$$

where χ is the dopant-site concentration, θ_dop_ is the local coverage at dopant sites, and Δ*Q*_dop_ is the corresponding charge transfer. A representative χ = 0.01 is assumed, consistent with experimentally realizable doping levels.^77^

The calculated variations in carrier concentration for SO_2_ and NO_2_ adsorption are presented in Figure 8c,d, respectively. For SO_2_, although doping produces modest improvements in site coverage, these enhancements do not translate into a meaningful modulation of the carrier concentration at the 1% doping level. Consequently, across all investigated systems, changes in δ*n* remain nearly identical, increasing approximately linearly with analyte concentration up to the 100 ppm range and reaching values on the order of −6 × 10^13^ e/cm^2^ at saturation (Figure 8c). This behavior indicates a comparable detection capacity for SO_2_ above 1 ppm regardless of the dopant type. Therefore, significantly higher dopant concentrations would be required to achieve noticeable performance gains—rendering such doping strategies generally impractical for improving MoS_2_ sensitivity toward SO_2_and, by extension, species such as CO_2_ and NH_3_.

In contrast, NO_2_ adsorption produces far more pronounced effects (Figure 8d). Even at a 1% dopant concentration, both Tc– and Re–MoS_2_ exhibit substantial carrier modulation at sub-ppm levels, with δ*n* exceeding −1 × 10^12^ e/cm^2^ at 0.001 ppm, suggesting ppb-level detection capability. This 2–4 order of magnitude increase relative to pristine MoS_2_ at low NO_2_ concentrations directly reflects the much larger charge transfer and coverage enhancements discussed earlier. The results confirm that n-type doping can strongly and selectively sensitize the monolayer toward NO_2_, making it far easier to detect and distinguish from other analytes such as SO_2_, which pristine MoS_2_ struggles to identify.^22^ This macroscopic trend is consistent with the DOS features obtained from the DFT calculations (see the Supporting Information). By comparison, p-type Nb–MoS_2_ exhibits a much weaker response, remaining nearly indistinguishable from the pristine surface.

## 4 Conclusions

This work establishes how the chemical identity of transition-metal dopants fundamentally governs the interaction landscape of MoS_2_ toward key gas molecules, linking microscopic adsorption characteristics with macroscopic sensing performance. By systematically examining dopants across groups 4, 5, 7, and 10 and adsorbates of distinct electronic character (NH_3_, CO_2_, SO_2_, and NO_2_), we show that TM substitution at the Mo site modulates adsorption primarily in a chemically selective rather than universal manner.

Structurally, TM dopants that themselves form stable TMD monolayers incorporate effectively into the MoS_2_ lattice upon substitution, consistent with reports of their successful integration via CVD synthesis. However, the resulting impact on adsorption depends strongly on the dopant type. p-Type dopants from groups 4 and 5 exhibit negligible influence on binding strength, while group 10 elements (Pd, Pt) alter local coordination and compromise environmental stability, rendering them unsuitable for sensing applications. In contrast, group 7 dopants (Tc and Re) provide a chemically coherent and structurally stable pathway to selectively enhance charge transfer and adsorption strength for electron-accepting species such as NO_2_, without affecting interactions with other gases.

At the macroscopic level, this selectivity translates directly into pronounced carrier concentration modulation and sub-ppm sensitivity to NO_2_ even at a modest 1% doping level—an improvement of several orders of magnitude over pristine MoS_2_. For other analytes (NH_3_, CO_2_, and SO_2_), doping effects remain minimal, indicating that TM substitution alone cannot universally tune MoS_2_ toward arbitrary targets.

Overall, the TM substitution of Mo in MoS_2_ emerges as a highly practical and scalable functionalization strategy capable of selectively enhancing electronic sensitivity to oxidizing analytes such as NO_2_. While its chemical diversity is inherently limited, this approach offers a powerful and manufacturable route to achieve both enhanced selectivity and reliable performance in real-world, room-temperature gas sensing.

## Supplementary Material



## Notes

(i) DOS plots for transition-metal-doped MoS_2_, (ii) comparison of charge transfer obtained from Löwdin and Bader population analysis methods, (iii) details of activation energy modeling for desorption, (iv) supercell size benchmarking for adsorption modeling in the dopant-dilute limit, and (v) DOS plots for selected adsorption systems (PDF)
