# Supplementary material for: First-Principles Design Rules for Selective Room-Temperature Gas Sensing in Transition-Metal-Doped MoS2
Source: ACS Appl Mater Interfaces. 2026 Feb 21;18(8):12938–48. doi: 10.1021/acsami.5c23014 (PMC13298819; doi:10.1021/acsami.5c23014)
Supplement: Supplementary file 1 [file am5c23014_si_001.pdf]

First-Principles Design Rules for Selective Room-Temperature Gas Sensing in Transition-Metal–Doped MoS_2

Maciej J. Szary[a,*]

[a] Institute of Physics, Poznan University of Technology, ul. Piotrowo 3, 61-138 Poznan, Poland

Supporting Information

Contents

1 Electronic Properties of Doped MoS_2 2

2 Löwdin and Bader Population Analysis 3

3 Modeling the Activation Energy of Desorption 4

4 Supercell Size Benchmarking for Adsorption Modeling in Dopant Dilute Limit 4

5 Impact of SO_2 and NO_2 Adsorption on the Electronic Properties of Doped MoS_2 5

* Corresponding author
Email address: maciej.szary@put.poznan.pl (Maciej J. Szary)

1. Electronic Properties of Doped MoS_2

The density of states (DOS) of transition-metal-doped MoS_2 monolayers is presented in Figure S1.

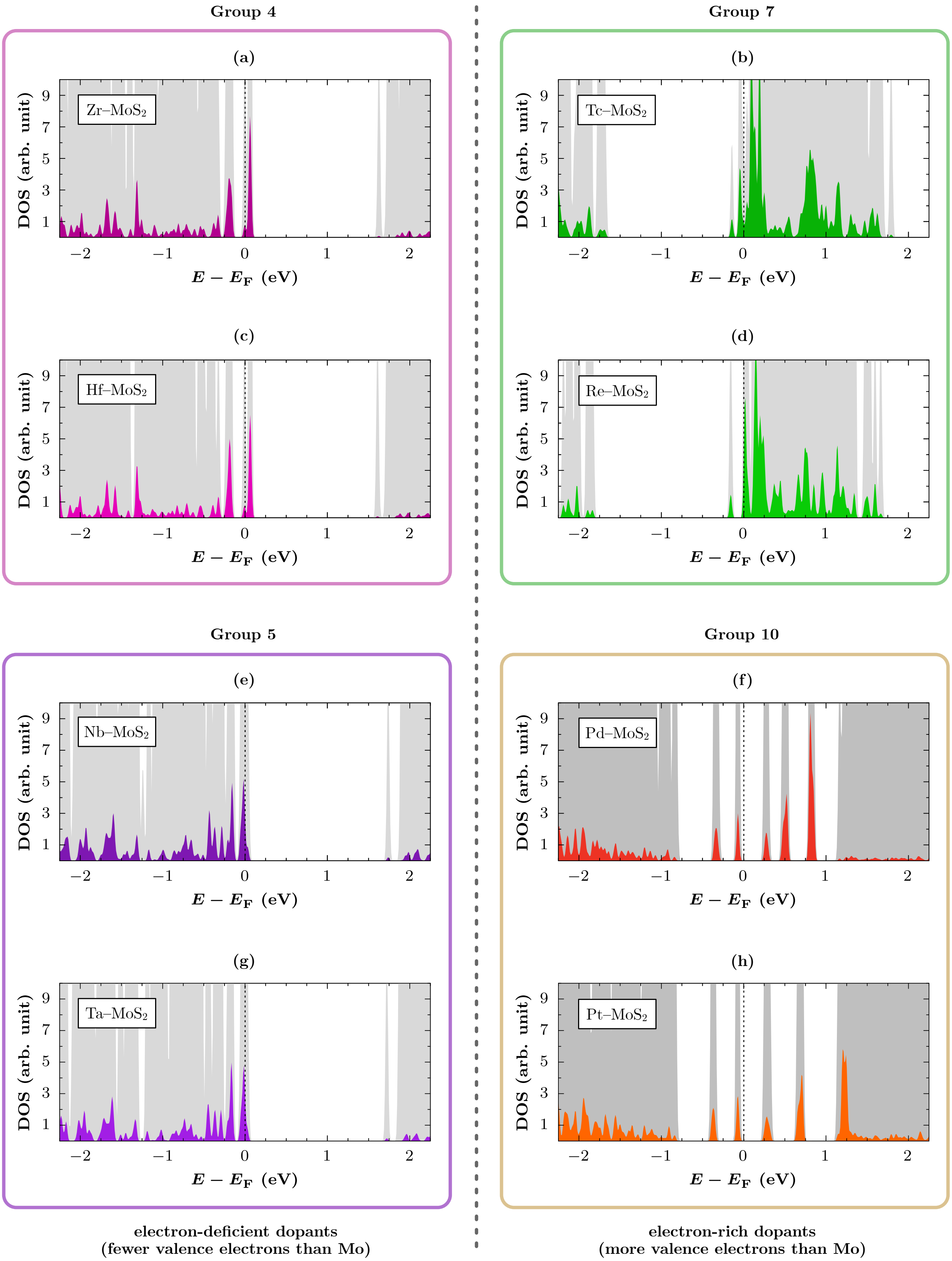

Figure S1: DOS plotted for (a) Zr-, (b) Tc-, (c) Hf-, (d) Re-, (e) Nb-, (f) Pd-, (g) Ta-, and (h) Pt-doped MoS_2 monolayers. Grey contours correspond to the total DOS, while the colored regions indicate the partial DOS contributions from the dopant *d* orbitals. The Fermi energy is denoted as E_F.

The effect of doping on the electronic structure of MoS_2 is primarily governed by the valence electron count of the dopant, showing pronounced differences across periodic groups but minimal variation within the same group.

Dopants from groups 4, 5, and 7 largely preserve the trigonal-prismatic coordination of the substituted Mo atom, and their electronic influence can therefore be rationalized by simple valence considerations. Group 4 elements (Zr, Hf) and group 5 elements (Nb, Ta) possess fewer valence electrons than Mo, leading to the formation of partially empty acceptor states near the valence-band edge of MoS_2 and resulting in consistent p-type behavior. Conversely, group 7 dopants (Tc, Re) have one additional valence electron relative to Mo, giving rise to partially filled donor states close to the conduction-band edge, consistent with n-type doping. These trends align well with available experimental observations for transition-metal-doped MoS_2 [1–5].

In contrast, group 10 dopants (Pd, Pt) do not maintain the trigonal-prismatic bonding environment of Mo and instead induce local lattice reconstruction. This structural distortion leads to a more complex modification of the electronic structure. Pd doping introduces five localized states within theMoS_2 band gap—two occupied and three unoccupied—situated near the Fermi level. A similar pattern is observed for Pt doping, although one of the unoccupied states lies near the conduction-band edge of MoS_2. These features indicate that group 10 dopants substantially perturb both the local geometry and the electronic landscape of the host lattice.

2. Löwdin and Bader Population Analysis

The primary population analysis method used to assess charge transfer throughout this study is the Löwdin scheme, as implemented in the Quantum ESPRESSO package. Because the Löwdin approach relies on the orthonormalization of atomic basis functions, it can exhibit sensitivity to the underlying basis set, occasionally leading to chemically inconsistent charge partitioning. To ensure the reliability of the results, we validated the Löwdin-derived charges against those obtained from Bader population analysis, which determines charge distribution directly from the real-space electron density topology and is therefore less dependent on basis-set choice.

Bader charges were computed using the Bader analysis code [6, 7]. The resulting charge transfers for the adsorption systems are summarized in Table S1 and compared with the Löwdin results. The two methods yield consistent chemical trends across all examined systems, with only minor quantitative differences. The average deviation between the Löwdin and Bader charge transfers is 0.0079 e, confirming excellent agreement.

Overall, the Bader analysis validates that the Löwdin method provides a robust and reliable means of quantifying charge transfer in doped TMD systems.

Table S1: Charge transfer (in $-e$) between gas molecules and pristine or transition-metal-doped MoS_2 monolayers obtained from Löwdin and Bader population analyses.

System	NH_3		CO_2		SO_2		NO_2	
	Löwdin	Bader	Löwdin	Bader	Löwdin	Bader	Löwdin	Bader
MoS_2	−0.0199	−0.0320	0.0143	0.0166	0.0525	0.0530	0.0442	0.0553
Zr–MoS_2	−0.0568	−0.0684	0.0134	0.0148	0.0504	0.0570	0.0317	0.0477
Hf–MoS_2	−0.0542	−0.0660	0.0138	0.0166	0.0582	0.0577	0.0340	0.0476
Nb–MoS_2	−0.0423	−0.0538	0.0144	0.0150	0.0529	0.0587	0.0325	0.0499
Ta–MoS_2	−0.0412	−0.0530	0.0137	0.0164	0.0575	0.0571	0.0316	0.0435
Tc–MoS_2	−0.0229	−0.0354	0.0148	0.0174	0.0844	0.0828	0.4278	0.4343
Re–MoS_2	−0.0208	−0.0338	0.0139	0.0175	0.1382	0.1337	0.4617	0.4666
Pd–MoS_2	−0.0314	−0.0447	0.0182	0.0223	0.1034	0.1025	0.0679	0.0796
Pt–MoS_2	−0.0053	−0.0184	0.0052	0.0200	0.0911	0.0897	0.0964	0.1097

3. Modeling the Activation Energy of Desorption

When modeling equilibrium coverage using the Langmuir adsorption model, it is essential to accurately describe the desorption kinetics, which are governed by the effective desorption barrier (E_{des}). This barrier is often approximated by the adsorption energy, such that $E_{des} = -E_{ads}$. This approach is computationally efficient and generally reasonable for weakly bound adsorbates, as it requires no additional calculations beyond geometry optimization.

However, molecular adsorption can in some cases noticeably influence both the zero-point energy (ZPE) and entropic contributions to the total free energy. To provide a more rigorous thermodynamic description, these terms can be explicitly included by using the Gibbs activation free energy for desorption ($\Delta G^{\ddagger}$) when determining the equilibrium coverage.

For this analysis, standard conditions are assumed: zero electrode potential, neutral pH, and a temperature of 300 K. Under these conditions, $\Delta G^{\ddagger}$ is defined as the free energy difference between the desorbed and adsorbed states, including contributions from the DFT electronic energy (E_{ads}), ZPE correction (ΔZPE), and vibrational entropy difference ($T\Delta S_{vib}$), along with the loss of one translational degree of freedom of the molecule upon adsorption ($\frac{1}{2}kT$). Thus, $\Delta G^{\ddagger}$ is given by:

$$\Delta G^{\ddagger} = E_{des} + \Delta \mathrm{ZPE} - T\Delta S_{vib} - \frac{1}{2}kT, \tag{1}$$

where $E_{des} = -E_{ads}$ represents the desorption energy obtained from DFT, ΔZPE is the difference in zero-point energy between the desorbed and adsorbed states, and $T\Delta S_{vib}$ is the corresponding entropic contribution.

The zero-point energy is computed as:

$$\mathrm{ZPE} = \sum_i \frac{1}{2} h\nu_i, \tag{2}$$

where h is Planck's constant and ν_i are the vibrational frequencies. The vibrational entropy (S_{vib}) is evaluated according to Ref. [8]:

$$S_{vib} = k \sum_i \left[\frac{h\nu_i}{kT\left(e^{\frac{h\nu_i}{kT}} - 1\right)} - \ln\left(1 - e^{-\frac{h\nu_i}{kT}}\right) \right], \tag{3}$$

where k is the Boltzmann constant and T is the temperature. The change in entropy, ΔS_{vib}, is obtained as the difference between the total vibrational entropy of the adsorbed system and the sum of the entropies of the isolated molecule and pristine substrate.

All vibrational frequencies were calculated using density functional perturbation theory (DFPT).

Within this framework, $\Delta G^{\ddagger}$ serves as a thermodynamic descriptor of the activation free energy for desorption and thus is always positive. After evaluation, $\Delta G^{\ddagger}$ is employed to determine adsorption site coverage in the Langmuir model.

It is important to note that, for the systems studied here, molecular adsorption induces minimal structural changes in either the adsorbate or the substrate. Consequently, ΔZPE and $T\Delta S_{vib}$ contributions remain small (below 30 meV), and $\Delta G^{\ddagger}$ is consistently dominated by the electronic DFT term.

4. Supercell Size Benchmarking for Adsorption Modeling in Dopant Dilute Limit

Selecting an appropriate supercell size for adsorption modeling requires balancing computational efficiency with physical accuracy—that is, choosing the smallest cell that adequately accommodates the adsorbate while minimizing undesired interactions between periodic images.

In the context of this study, this consideration is particularly critical, since the DFT-derived adsorption parameters—obtained using a 4×4 supercell corresponding to a 6.25% dopant concentration—are subsequently employed to model molecular coverage and macroscopic response at much lower, experimentally relevant doping levels ($\chi = 1\%$). Therefore, the DFT model must faithfully represent the dilute limit, where adsorption parameters become effectively insensitive to dopant–dopant separation.

To assess convergence with respect to supercell size, benchmark tests were performed for SO_2@Re-MoS_2 and NO_2@Re–MoS_2, as these systems exhibit the strongest adsorption among the studied gases and are thus expected to be most sensitive

to periodic effects.

Adsorption energy (E_{ads}) and charge transfer (ΔQ) were evaluated using supercells ranging from 2×2 to 7×7 (see Figure S2). The results show consistent convergence behavior for both molecules and parameters. Significant variations are observed only for the smallest cells (2×2 and 3×3), whereas beyond the 4×4 supercell, size effects are largely saturated. Specifically, at the 4×4 level, E_{ads} and ΔQ reach approximately 250 meV and 0.14 e for SO_2, and 600 meV and 0.45 e for NO_2, respectively.

Comparison with the largest tested system confirms that finite-size effects are minimal. For the 7×7 supercell, the NO_2 adsorption energy is only 2.1% higher than in the 4×4 case, while for SO_2 the deviation is just 1.5%. Similarly, the corresponding differences in ΔQ are 2.7% and 1.6%, respectively.

These results confirm that a 4×4 supercell provides an accurate and computationally efficient representation of the dilute doping regime, suitable for evaluating adsorption energetics and charge transfer in doped MoS_2 monolayers.

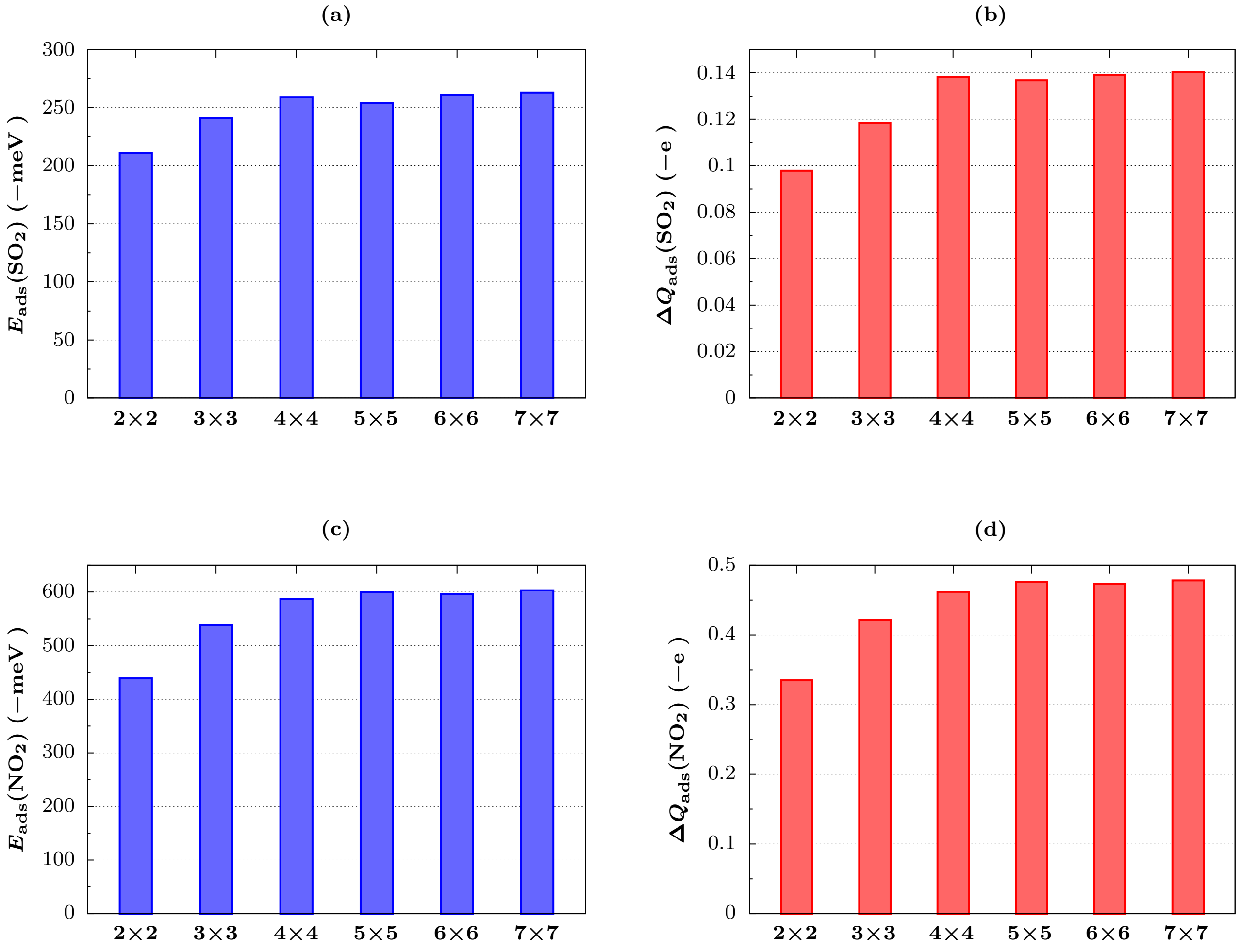

Figure S2: (a) Adsorption energy and (b) charge transfer for SO_2 adsorption on Re–MoS_2 evaluated using supercells ranging from 2×2 to 7×7. (c) Adsorption energy and (d) charge transfer for NO_2 adsorption on Re–MoS_2 evaluated using supercells ranging from 2×2 to 7×7.

5. Impact of SO_2 and NO_2 Adsorption on the Electronic Properties of Doped MoS_2

Although direct interpretation of DFT results may be limited in quantitative accuracy—since the computational models do not fully reproduce experimental conditions—the DOS analysis nonetheless provides valuable qualitative insights into how gas adsorption modifies the electronic structure of doped MoS_2. Figure S3 compares the DOS for two representative adsorbates, SO_2 and NO_2, on doped systems that exhibit distinct adsorption behaviors: Nb, which shows minimal adsorption enhancement, and Tc and Re, which induce moderate and strong adsorption effects.

For Nb-doping, both SO_2 (Figure S3a) and NO_2 (Figure S3b) exert a negligible influence on the electronic structure of the monolayer. The total DOS and the dopant *d*-orbital contributions near the Fermi level remain largely unchanged, with any new states observed within the MoS_2 band gap arising primarily from the molecular orbitals of the adsorbate rather than the substrate.

In contrast, SO_2 adsorption on Tc- and Re-doped monolayers (Figures S3c and S3e) produces only minor perturbations in the DOS, suggesting that charge transfer in these systems is primarily due to localized polarization effects. This behavior is consistent with the macroscopic carrier-modulation model, which predicts limited sensing enhancement for SO_2.

NO_2 adsorption, however, has a pronounced effect on the electronic structures of Tc- and Re-doped MoS_2 (Figures S3d and S3f). The interaction leads to a noticeable redistribution of the dopant-derived states, a downward shift of the donor levels from the conduction-band edge into the band gap, and an overall Fermi-level shift of approximately 0.5 eV. These features indicate strong electronic coupling between the adsorbate and the doped substrate and the overall loss of free electrons carriers, supporting the observed enhancement in NO_2 sensitivity predicted by the carrier-modulation analysis.

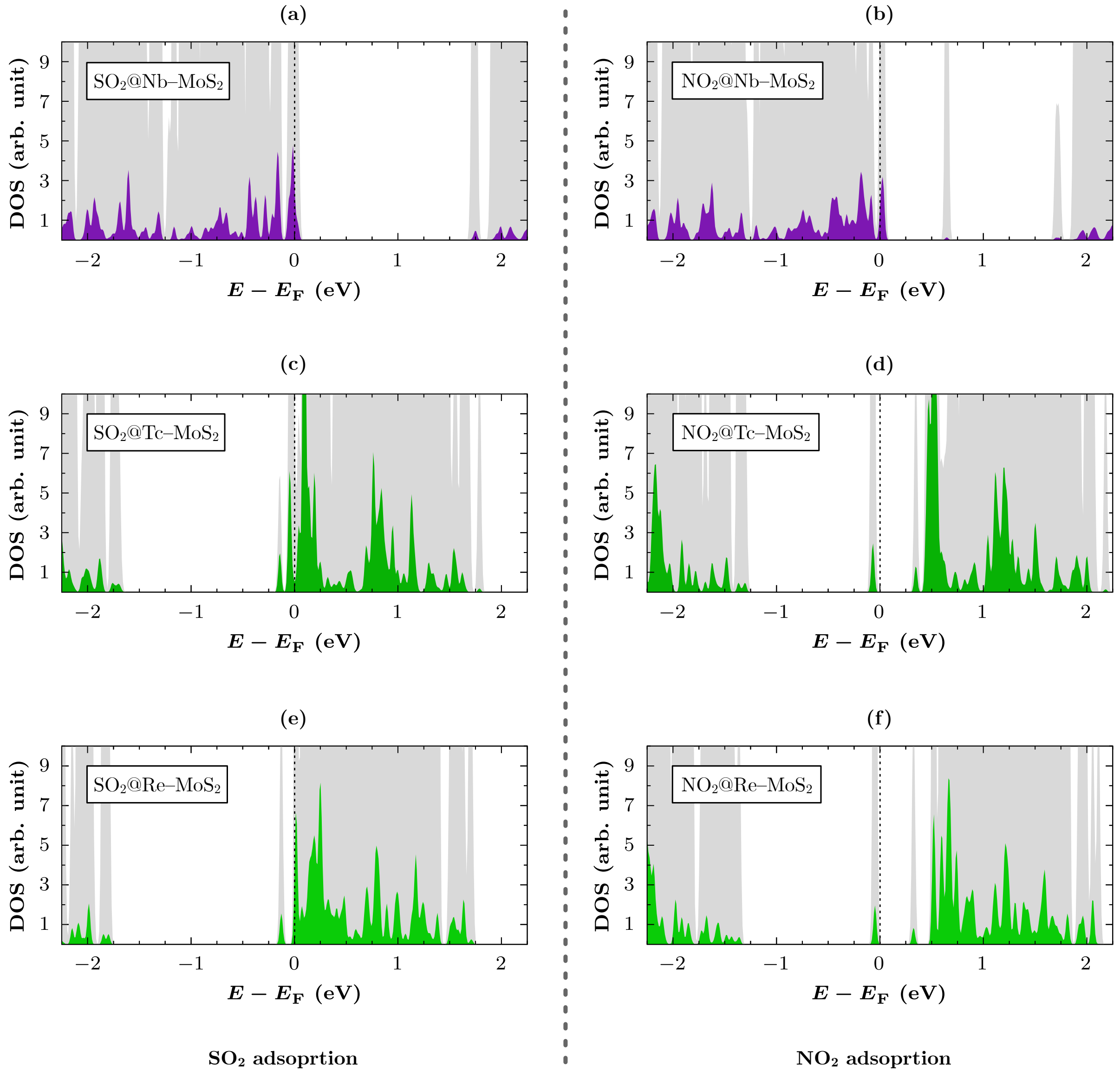

Figure S3: DOS plots for (left) SO_2 and (right) NO_2 adsorption on (a,b) Nb-, (c,d) Tc-, and (e,f) Re-doped MoS_2. Grey contours correspond to the total DOS, while the colored regions indicate the partial DOS contributions from the dopant *d* orbitals. The Fermi energy is denoted as E_F.

References

[1] M. R. Laskar, D. N. Nath, L. Ma, I. Lee, Edwin W., C. H. Lee, T. Kent et al, p-type doping of MoS_2 thin films using Nb, Applied Physics Letters 104 (9) (2014) 092104. doi:10.1063/1.4867197.

[2] T. Hallam, S. Monaghan, F. Gity, L. Ansari, M. Schmidt, C. Downing et al, Rhenium-doped MoS_2 films, Applied Physics Letters 111 (20) (2017) 203101. doi:10.1063/1.4995220.

[3] M. Li, J. Yao, X. Wu, S. Zhang, B. Xing, X. Niu et al, P-type doping in large-area monolayer MoS_2 by chemical vapor deposition, ACS Applied Materials & Interfaces 12 (5) (2020) 6276–6282. doi:10.1021/acsami.9b19864.

[4] A. Raza, U. Qumar, A. Haider, S. Naz, J. Haider, A. Ul-Hamid et al, Liquid-phase exfoliated MoS_2 nanosheets doped with p-type transition metals: a comparative analysis of photocatalytic and antimicrobial potential combined with density functional theory, Dalton Trans. 50 (2021) 6598–6619. doi:10.1039/D1DT00236H.

[5] M. Li, X. Wu, W. Guo, Y. Liu, C. Xiao, T. Ou et al, Controllable p-type doping of monolayer MoS_2 with tantalum by one-step chemical vapor deposition, J. Mater. Chem. C 10 (2022) 7662–7673. doi:10.1039/D2TC01045C.

[6] W. Tang, E. Sanville and G. Henkelman, A grid-based bader analysis algorithm without lattice bias, Journal of Physics: Condensed Matter 21 (8) (2009) 084204. doi:10.1088/0953-8984/21/8/084204.

[7] M. Yu and D. R. Trinkle, Accurate and efficient algorithm for bader charge integration, The Journal of Chemical Physics 134 (6) (2011) 064111. doi:10.1063/1.3553716.

[8] G. Brehm, M. Reiher and S. Schneider, Estimation of the vibrational contribution to the entropy change associated with the low- to high-spin transition in $Fe(phen)_2(NCS)_2$ complexes: Results obtained by IR and Raman spectroscopy and DFT calculations, The Journal of Physical Chemistry A 106 (50) (2002) 12024–12034. doi:10.1021/jp026586o.
